# Competing Factors Link to Bone Health in Polycystic Ovary Syndrome: Chronic Low-Grade Inflammation Takes a Toll

**DOI:** 10.1038/s41598-017-03685-x

**Published:** 2017-06-13

**Authors:** Shirin Kalyan, Millan S. Patel, Elaine Kingwell, Hélène C. F. Côté, Danmei Liu, Jerilynn C. Prior

**Affiliations:** 1 Centre for Menstrual Cycle and Ovulation Research, Division of Endocrinology, Department of Medicine, University of British Columbia; Vancouver Coastal Health Research Institute, Vancouver, Canada; 2 Women’s Health Research Institute, Vancouver, Canada; 3 Deptartment of Medical Genetics, University of British Columbia, Vancouver, Canada; 4 Division of Neurology, Department of Medicine, University of British Columbia, Vancouver, Canada; 5 Department of Pathology and Laboratory Medicine, University of British Columbia, Vancouver, Canada; 6 Centre for Hip Health and Mobility, University of British Columbia, Vancouver, Canada; 7 School of Population and Public Health, University of British Columbia, Vancouver, Canada

## Abstract

Chronic inflammation predisposes to poor bone health. Women with polycystic ovary syndrome (PCOS) experience androgen excess, ovulatory disturbances, insulin resistance, abdominal adiposity and chronic inflammation. Our objective was to investigate the relationships among bone health parameters, chronic subclinical inflammation and anthropometric measures in premenopausal women with and without PCOS. In 61 premenopausal women, 22 women with PCOS and 39 controls, we assessed bone parameters (total hip bone mineral density [BMD] by dual-energy X-ray absorptiometry and radius strength-strain index [SSI] by peripheral quantitative computed tomography), inflammation (C-reactive protein/albumin), oxidative stress (leukocyte telomere length, urinary 8-hydroxydeoxyguanosine); hemoglobin A1c; anthropometric measures (body mass index, waist-to-height ratio, cross-sectional muscle area). A diagnosis of PCOS negatively predicted (beta = −0.251, *p* = 0.022) hip BMD in a regression model including weight. In women with PCOS, inflammation, which was predicted by increased waist-to-height ratio and current use of oral contraceptives, attenuated the positive influences of increased weight and muscle mass on bone strength and was inversely associated with radial SSI (R^2^ = 0.25, *p* = 0.018). In conclusion, chronic subclinical inflammation may negatively impact bone physiology in women with PCOS. Strategies focused on reducing abdominal adiposity and avoiding medications that increase inflammation may counter this effect.

## Introduction

Polycystic ovary syndrome (PCOS), a complex endocrine disorder characterized by androgen excess, menstrual irregularities and ovulatory disturbances, is often associated with obesity (particularly abdominal adiposity) and insulin resistance^1–4^. It is currently considered the most common reproductive disorder, affecting 5–15% of premenopausal women worldwide^3, 5^. Women with PCOS are at an increased risk for a number of health problems, including infertility, cardiovascular disease and diabetes^1^. However, hyperandrogenemia, hyperinsulinemia and the tendency to increased body mass index (BMI) are collectively thought to protect women with PCOS against bone fragility disorders such as osteoporosis. Consequently, women with PCOS are generally believed to have a reduced risk for fractures^1, 6, 7^.

The adverse effects of chronic inflammation and oxidative stress on bone remodeling and quality are increasingly recognized^8–11^. In addition to endocrine dysfunction, women with PCOS are found to be at greater risk for chronic subclinical inflammation^12, 13^ and potentially experience more oxidative stress^14^. In this investigation, we sought to determine whether exposure to chronic subclinical inflammation and oxidative stress may compromise optimal bone strength and quality in women with PCOS despite their greater weight, higher levels of androgens and greater likelihood for elevated insulin levels. Specifically, we assessed the total hip areal bone mineral density (BMD) by dual-energy X-ray absorptiometry (DXA) as well the architectural bone parameters contributing to the strength-strain index (SSI) of the radius by peripheral quantitative computed tomography (pQCT) in premenopausal women with and without PCOS. The radial SSI provides a calculated, objective measure of bone strength and quality that is independent of the influence of body weight on bone size, which is a frequent confounder of studies using only areal BMD. We subsequently determined how inflammation, oxidative stress, body mass indices and a diagnosis of PCOS contributed to shaping the bone health characteristics investigated.

## Materials and Methods

### Study participants

Premenopausal women between the ages of 35 and 47 from the Vancouver region (Canada) were invited to participate through advertisements placed in community newspapers, on notice boards, on our website (www.cemcor.ca), and by referrals through professional networks as previously described^15^. This age range was selected as women would be expected to have already achieved peak bone mass, and the effects of inflammation, oxidative stress and cellular aging would be more clearly apparent, if present. There were no restrictions with respect to weight or use of various therapies, including oral contraceptives; however, current smoking was an exclusion criterion due to its effects on inflammation and oxidative stress^16, 17^. A diagnosis of PCOS was based on the recommendations by the National Institute of Health criteria supported by the Androgen Excess and PCOS Society^18^ which requires evidence of androgen excess and a history of amenorrhea/oligomenorrhea with no alternate explanation(s). Premenopausal women with no history of amenorrhea/oligomenorrhea in the context of androgen excess (“controls”) were similarly recruited.

All women provided written informed consent and had their anthropometric measures and blood samples taken during their clinic visit. Women were assessed to ensure there were no signs of fever, infection or other acute illness. The study was approved by the Clinical Research Ethics Board of the University of British Columbia (H10-02987) and was performed within the tenets of the Declaration of Helsinki for research involving human subjects.

### Data availability

Data that has been published and does not compromise participant confidentiality may be made upon available to academic researchers upon request.

#### Areal Bone Mineral Densitometry

The left hip was scanned by DXA using a single Hologic QDR4500 machine (Hologic Inc., Waltham, MA, USA) on array mode, and all the DXA scans were analyzed using the standard Hologic analysis protocol. The total hip and femoral neck bone mineral density (BMD; g/cm^2^) and calculated Z-scores matched for age, weight (for women between 25–100 kg) and ethnicity are reported.

#### Peripheral Quantitative Computed Tomography (pQCT)

The peripheral QCT Stratec XCT 3000 densitometer (Stratec Medizintechnic GmbH, Pforzheim, Germany) was used to acquire a single 2.0+/−0.5 mm slice of the non-dominant forearm at the 30% site (proximal to the medial corner of the distal end plate of the radius). If a previous fracture had been experienced on the non-dominant arm, the dominant arm was scanned. We used a voxel size of 0.3 mm and a scan speed of 30 mm/s. A 30 mm planar scout view over the wrist joint line was used to define the anatomic reference line. The pQCT scans were analyzed by XCT 6.0 software using Cort mode 1 (710 mg/cm^3^) as the threshold for cortical bone. In a separate analysis, Cort mode 1 (480 mg/cm^3^) was used to calculate the polar strength-strain index (SSI, mm^3^) which provides an estimate of torsional bone strength, as previously described^19^. Contour mode 3 (40 mg/cm^3^), Peel mode 2 (40 mg/cm^3^), and Cort mode 1 (710 mg/cm^3^) with filter F03F05F05 were used to assess muscle cross-sectional area (MCSA = [muscle + bone − bone] area, mm^2^), an estimate of muscle strength^20^. Muscle density (=[muscle + bone − bone] mass/[muscle + bone − bone] area) was calculated using Contour mode 1 (280 mg/cm^3^), Peel mode 2 (40 mg/cm^3^), and Cort mode 1 (480 mg/cm^3^) was used to assesses fat within the muscle compartment. The reported bone structural variables include total bone cross-sectional area, cortical area, cortical thickness from a circular ring model^21^, SSI and both the total and cortical density. Measurements were made by one of two trained operators and all scans were analyzed by a single expert.

#### Biochemical assays

Blood hemoglobin A1c (HbA1c), serum C-reactive protein (CRP), serum albumin, and urinary creatinine were measured by chemiluminescence on a Dimension Vista® System (Siemens, Oakville, Canada) using Flex® reagent cartridges (K3105A, K7046, K1013, K1033, respectively). The cartridge used for the CRP analysis employed the CardioPhase® high sensitivity CRP method. The OxiSelect^TM^ Oxidative DNA Damage Enzyme-Linked Immunosorbent Assay (ELISA) Kit (Cell Biolabs, Inc., Cedarlane, Burlington, Canada) was used to quantify urinary 8-hydroxydeoxyguanosine (8-OHdG) as a measure of oxidative stress.

#### Telomere Length Analysis

Relative leukocyte telomere length, a measure of cellular aging and a proxy for oxidative damage^22^, was assessed by a multiplex quantitative PCR (qPCR) assay^23^. The assay is based on measuring the telomere length signal (T) in leukocyte DNA relative to that of a single copy gene (S), in this case albumin, to yield relative T/S ratios that are proportional to the average telomere length. The assay was performed using a LightCycler® 480 (Roche Applied Science, Laval, Canada) as previously described^24^, with the following modifications: the SYBR® Select Master Mix (Applied Biosystems®, Life Technologies^TM^, Burlington, Canada) was used and the standard curve was generated by serial dilutions (1:3) of pooled human buffy coat DNA ranging from 823 to 200,000 copies of albumin (S) and 5 to 1134 relative copies of T (corresponding to a total DNA concentration of ~18 to 142 ng/µL).

#### Statistical Analysis

All variable distributions were inspected visually and assessed using the D’Agostino-Pearson omnibus normality test. CRP was among the variables with non-normal distribution and was therefore log transformed for inclusion in the linear regression models. Descriptive statistics are provided as mean ± standard deviation (SD) and the 95% confidence interval of the means were noted. The two-sided t-test or the Mann-Whitney U-test were used to compare group differences with statistical significance set at α = 0.05. Associations between variables were explored separately in women with and without PCOS using Spearman’s rank correlation. The index for inflammation was estimated using the log transformed CRP/albumin ratio to capture the strong inverse relationship between these two proteins under inflammatory states^25^. Both CRP and albumin are liver-derived, the former is a positive acute phase factor and the latter a negative acute phase factor (*i*.*e*. levels of albumin are reduced under states of inflammation)^25^. Abdominal adiposity was estimated using the waist-to-height ratio as it correlates more strongly with cardio-metabolic risk factors than doe waist circumference or BMI alone^26^. Linear regression models were used to assess the individual contribution of independent variables in predicting the primary bone outcomes: total hip BMD (by DXA) and the radius SSI (by pQCT), in women with PCOS and controls. Multiple linear regression models that included all subjects (PCOS and controls) were also built for the two primary bone outcomes and included a diagnosis of PCOS, weight, and log(CRP/albumin) as independent variables of interest. Potential effect modification between PCOS and log(CRP/albumin) was assessed by inclusion of an interaction term and further exploration was done with stratified analyses. As a secondary analysis, a multiple regression model for log(CRP/albumin) was built that included a diagnosis of PCOS, waist-to-height ratio, and current oral combined hormonal contraceptive (CHC) use as independent variables. Data were analyzed using SPSS version 21.00 (SPSS Inc., Chicago, IL, USA) and graphed using Prism GraphPad (version 5.00 for Windows, GraphPad Software, San Diego, CA, USA).

## Results

### Characteristics of premenopausal women with PCOS and controls

Forty-one control women and 25 women with PCOS initially enrolled in the study (Table 1). Of these 66 women, 61 attended bone assessment appointments (39 controls; 22 with PCOS). The rate of current CHC use was 15% in control women (n = 6) and 18% in those with PCOS (n = 4). The presence of inflammation (as assessed by CRP and the CRP/albumin ratio) was significantly greater in women with PCOS compared to controls. There was no statistical difference between groups in relative leukocyte telomere length (LTL) or urinary 8-OHdG levels, although control women were older (42.5 ± 4.2 vs 40.3 ± 3.4 y). Despite women with PCOS having significantly greater BMI and non-dominant forearm muscle cross-sectional area (MCSA), their unadjusted bone density and strength parameters were not greater than control women (Table 2).

**Table 1:** Characteristics of premenopausal women with and without polycystic ovary syndrome (PCOS).

	Controls n = 41^§^		PCOS n = 25		*p value*
	Mean ± SD	(95% CI)	Mean ± SD	(95% CI)	
Age (y)	42.5 ± 4.2	(41.2, 43.9)	40.3 ± 3.4	(38.9, 41.7)	**0**.**029**
Weight (kg)	67.0 ± 14.6	(62.4, 71.6)	81.6 ± 21.8	(72.6, 90.6)	**0**.**002**
Height (cm)	163.6 ± 8.1	(161.0, 166.1)	166.2 ± 6.3	(163.6, 168.8)	0.161
BMI (kg/m^2^)	25.1 ± 4.6	(23.6, 26.5)	29.7 ± 7.9	(26.4, 32.9)	**0**.**003**
Waist Circumference (WC; cm)	79.4 ± 10.5	(76.1, 82.7)	89.3 ± 15.3	(83.0, 95.7)	**0**.**010**
Waist-to-height ratio	0.49 ± 0.06	(0.47, 0.51)	0.54 ± 0.10	(0.50, 0.58)	**0**.**022**
**Inflammatory & Metabolic Markers**					
C-reactive Protein (CRP) (mg/L)	1.47 ± 1.54	(0.98, 1.96)	2.85 ± 3.21	(1.52, 4.17)	**0**.**023**
Albumin (g/L)	39.68 ± 2.38	(38.91, 40.44)	38.88 ± 2.95	(37.66, 40.10)	0.237
CRP/albumin	37.93 ± 41.83	(24.55, 51.31)	76.93 ± 90.00	(39.78, 114.10)	**0**.**021**
HbA1c	5.36 ± 0.42	(5.22, 5.49)	5.31 ± 0.42	(5.14, 5.49)	0.669
**Oxidative Stress Measures**					
8-OHdG/creatinine (pg/mmol)	5.52 ± 3.27	(4.46, 6.5)	5.18 ± 2.67	(4.08, 6.28)	0.504
Relative leukocyte telomere length	5.13 ± 0.36	(5.02, 5.25)	5.18 ± 0.37	(5.03, 5.34)	0.483

^§^One woman was unable to give blood, n = 40 for CRP, albumin and HbA1c.

**Table 2:** Bone and muscle parameters of premenopausal women with polycystic ovary syndrome (PCOS) and controls.

	Controls n = 39^		PCOS n = 22*		*p value*
	Mean ± SD	(95% CI)	Mean ± SD	(95% CI)	
**DXA**					
Total Hip (g/cm^2^) aBMD	0.97 ± 0.11	(0.93, 1.00)	0.96 ± 0.13	(0.91, 1.02)	0.963
Femoral Neck (g/cm^2^) aBMD	0.84 ± 0.12	(0.80, 0.88)	0.81 ± 0.11	(0.76, 0.86)	0.403
Femoral Neck aBMD Z score	0.30 ± 1.08	(−0.05, 0.65)	−0.03 ± 1.03	(−0.50, 0.44)	0.262
Total Hip aBMD Z score	0.44 ± 0.93	(0.14, 0.74)	0.37 ± 1.08	(−0.12, 0.86)	0.794
**pQCT – Radius 30% site**					
Cortical BMD (mg/cm^3^)	1260.0 ± 16.7	(1254.0, 1265.0)	1255.0 ± 25.7	(1244.0, 1267.0)	0.494
Total area (mm^2^)	90.7 ± 12.4	(86.7, 94.8)	95.6 ± 11.8	(90.3, 100.8)	0.143
Cortical area (mm^2^)	74.9 ± 8.4	(72.2, 77.6)	76.8 ± 6.1	(74.1, 79.5)	0.362
Cortical thickness (mm)	3.17 ± 0.32	(3.06, 3.27)	3.10 ± 0.18	(3.02, 3.17)	0.259
Strength-Strain Index (SSI, mm^3^)	213.6 ± 41.0	(200.3, 226.9)	228.8 ± 38.5	(211.8, 245.9)	0.169
Muscle cross-sectional area (MCSA; mm^2^)	1470.0 ± 206.1	(1400.0, 1541.0)	1643.0 ± 265.3	(1523.0, 1764.0)	**0.009**
Muscle density (mm^3^)	77.9 ± 5.9	(75.8, 79.9)	76.5 ± 3.7	(74.9, 78.2)	0.309

^4 women failed the muscle analysis due to movement artifacts.

*1 woman was over the weight limit for the DXA hip scan (n = 21); 1 woman failed the muscle analysis due to movement artifact (n = 21).

### Predictors of total hip bone density by DXA in women with PCOS and controls

**Figure 1:**
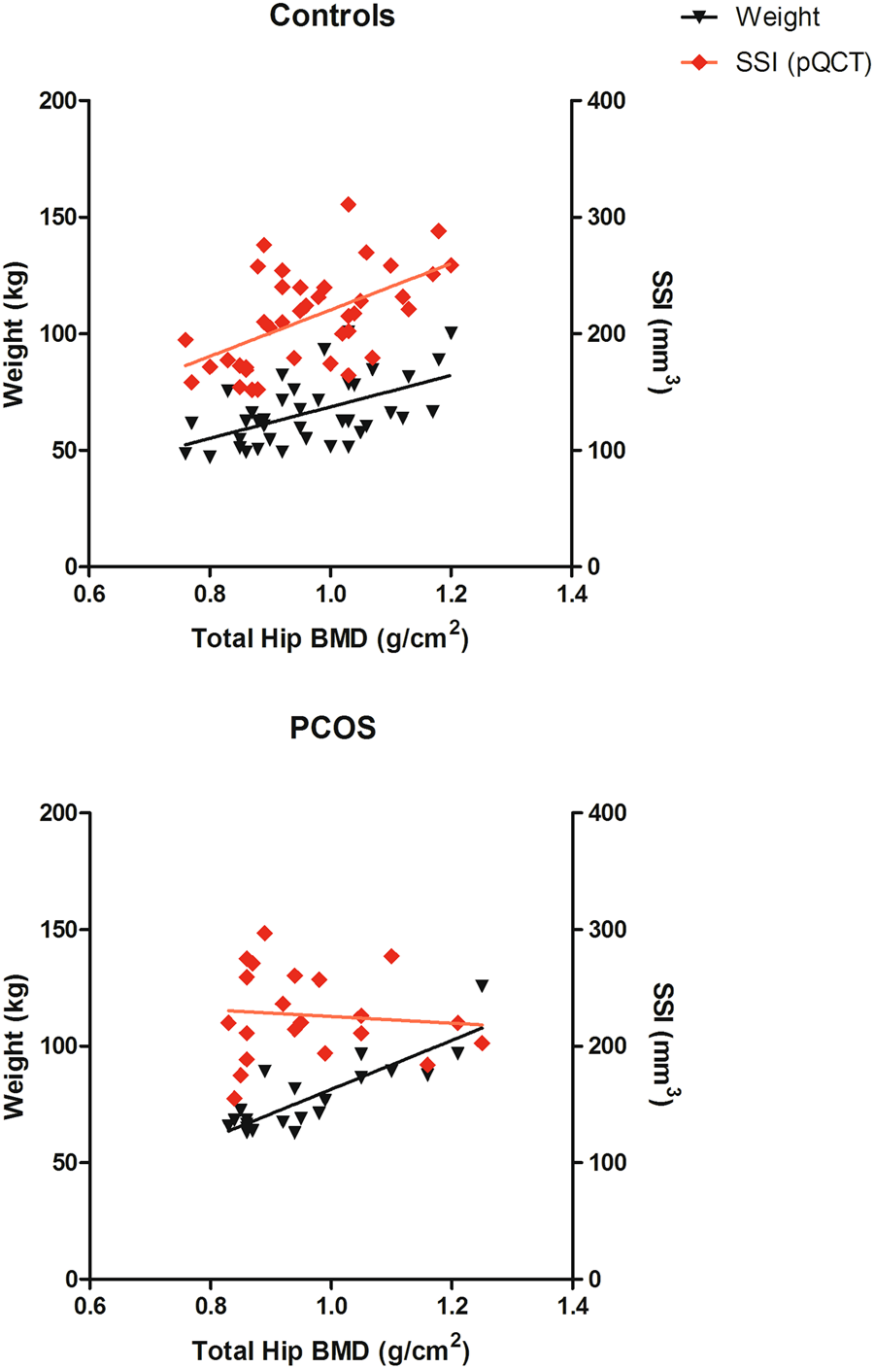
Linear regression of the total hip BMD (g/cm^2^) with weight (kg) and the radial strength-strain index (SSI, mm^3^) as the independent variables for women with PCOS (n = 21) and controls without PCOS (n = 39). Weight was a significant predictor of the total hip BMD, R^2^ = 0.72, *p* < 0.0001 for women with PCOS; R^2^ = 0.29, *p* < 0.001 for controls. The radial SSI was not a significant predictor of the total hip BMD for the women with PCOS, R^2^ = 0.01, *p* = 0.68, but was important in the controls, R^2^ = 0.30, *p* < 0.001.

There were significant correlations between increased weight, and greater waist circumference, and the total hip BMD (Table 3). Among control women, increased height and increased radial SSI were significantly related to the total hip BMD; these relationships were not seen in women with PCOS (Table 3). Linear regression analysis showed that weight and the radial SSI individually predicted 29% (*p* < 0.001) and 30% (*p* < 0.001) of the variance in hip BMD for the controls, respectively (Fig. 1, top panel). For women with PCOS, although weight predicted 71% (*p* < 0.0001) of the variance in hip BMD, it was uncoupled from radial SSI, which showed no significant predictive power (R^2^ = 0.009, *p* = 0.68) (Fig. 1, bottom panel). The oxidative stress measures did not associate with hip BMD in either group.

**Table 3:** Correlations* among total hip bone mineral density (BMD, g/cm^2^) and the radial strength-strain index (SSI, mm^3^) by peripheral quantitative computed tomography site in women with polycystic ovary syndrome (PCOS) and controls.

	Controls (n = 39)				PCOS (n = 22)			
	Total Hip BMD Spearman r	*p value*	Radius SSI Spearman r	*p value*	Total Hip BMD Spearman r	*p value*	Radius SSI Spearman r	*p value*
Weight (kg)	**0**.**531**	**0**.**001**	**0**.**400**	**0**.**012**	**0**.**730**	**0**.**000**	−0.024	0.917
Height (cm)	**0**.**406**	**0**.**011**	**0**.**483**	**0**.**002**	0.078	0.736	**0**.**560**	**0**.**007**
BMI (kg/m^2^)	0.304	0.063	0.151	0.367	**0**.**609**	**0**.**003**	−0.164	0.465
Waist circumference (cm)	**0**.**399**	**0**.**012**	0.205	0.211	**0**.**685**	**0**.**001**	−0.086	0.702
Waist-to-height ratio	0.230	0.164	0.033	0.846	**0**.**519**	**0**.**016**	−0.243	0.275
Radius SSI (mm^3^, pQCT)	**0**.**581**	**0**.**000**			0.061	0.792		
Radius MCSA (mm^2^)^	0.243	0.136	**0**.**523**	**0**.**001**	0.020	0.935	0.143	0.536
8-OHdG/creatinine (pg/mmol)	0.002	0.989	0.187	0.268	−0.154	0.506	0.027	0.907
Relative leukocyte telomere length	−0.004	0.980	0.118	0.473	0.272	0.233	−0.090	0.689

*Data reported are Spearman ranked correlations with two-tailed p values.

^4 controls and 1 woman with PCOS failed the muscle cross-sectional area (MCSA) analysis due to movement artifacts.

In a multivariate linear regression model for the total hip BMD of the entire study cohort (n = 60), a diagnosis of PCOS was negatively related to total hip BMD (β = −0.25, *p* = 0.022) when weight was included; adjusted R^2^ = 0.40, *p* < 0.001 for the model.

### Predictors of Radial Strength-Strain Index in women with PCOS and controls

There were clear divergences between women with and without PCOS with respect to factors associated with radial SSI. Weight, height, and the forearm MCSA were all positively associated with SSI in the controls (Table 3). However, only height was positively associated with radial SSI in women with PCOS, with no influence of either weight or MCSA.

**Figure 2:**
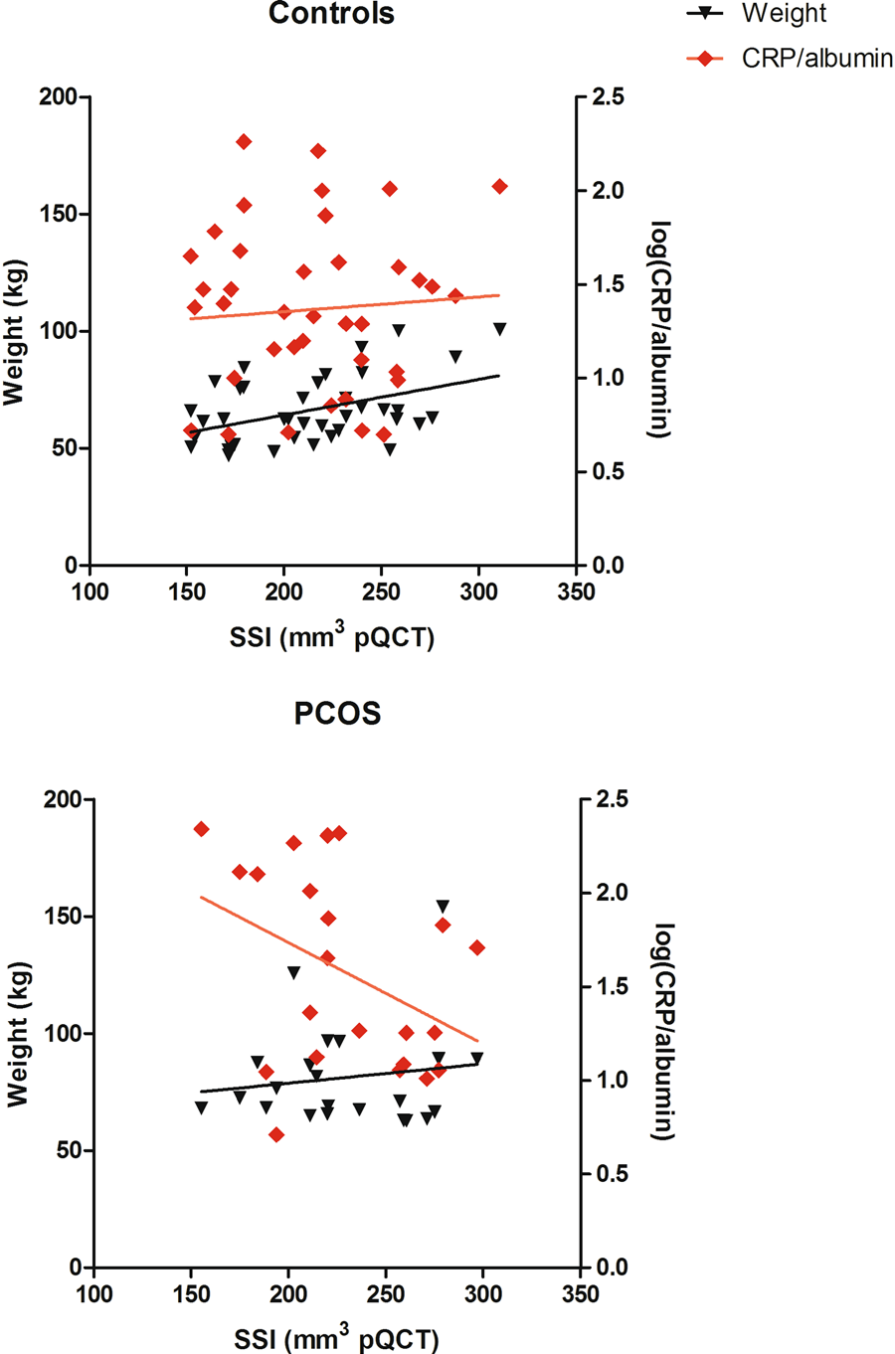
Linear regression of the radius strength-strain index (SSI, mm^3^) including weight (kg) and log(CRP/albumin) as an index of inflammation as the independent variables for women with PCOS (n = 22) and controls (n = 39). Weight was not a significant predictor of the radial SSI in women with PCOS, R^2^ = 0.02, *p* = 0.53, but was important in the controls, R^2^ = 0.19, *p* = 0.006. Log(CRP/albumin) was predictive of the SSI in women with PCOS, R^2^ = 0.25, *p* = 0.02, but was not for women without PCOS, R^2^ < 0.01, *p* = 0.66.

Linear regression analysis shows that inflammation (CRP/albumin) is adversely related to radial SSI in women with PCOS (R^2^ = 0.25, *p* = 0.018), (Fig. 2, bottom panel), but not in control women (Fig. 2, top panel). Rather weight (R^2^ = 0.19, *p* = 0.006) and MCSA (R^2^ = 0.19, *p* = 0.011) were the primary predictors of radial SSI in women without PCOS.

Multivariate linear regression for radial SSI (n = 61) including weight, log(CRP/albumin), and diagnosis of PCOS confirmed that both weight (β = 0.431, *p* = 0.004) and log(CRP/albumin) (β = −0.272, *p* = 0.048) were significant predictors in the main effects model (adjusted R^2^ = 0.13, *p* = 0.011). However, as illustrated in Fig. 2, there was a significant interaction effect (β = −0.858, *p* = 0.043 for PCOS * log(CRP/albumin) as the interaction term) between PCOS and log(CRP/albumin), whereby increased log(CRP/albumin) was significantly associated with decreased SSI only in the PCOS group.

### Predictors of subclinical inflammation [log(CRP/albumin)]

In light of the strong negative relationship between inflammation, as measured by the CRP/albumin ratio, and radial SSI as a measure of bone strength in women with PCOS, we explored variables that may predict high levels of inflammation. Abdominal adiposity is a known contributor to chronic subclinical inflammation and increased serum CRP levels^27^; oral estrogen therapy is also associated with elevated CRP levels^25^. A total of 10 women (15% of controls; 18% of those with PCOS) were taking CHC at the time of the study. Log(CRP/albumin) levels differed between current CHC users (1.9 ± 0.4) versus non-users (1.4 ± 0.4), *p* = 0.01. A multivariate regression model for log(CRP/albumin) levels including waist-to-height ratio (a measure of central adiposity), current use of CHC and a diagnosis of PCOS suggested that log(CRP/albumin) was independently predicted by the waist-to-height ratio (β = 0.6, *p* < 0.001) and current CHC use (β = 0.4; *p* < 0.001), but not directly by PCOS diagnosis (β = −0.015, *p* = 0.89); adjusted R^2^ = 0.42, *p* < 0.001 for the model.

## Discussion

Chronic low-grade inflammation is a major inducer of osteoclast differentiation thereby increasing bone resorption. Thus, people living with health conditions associated with chronic inflammation, such as inflammatory bowel disease, periodontitis, psoriasis and rheumatoid arthritis, are found to experience accelerated bone loss and have a heightened risk of osteoporosis^28–30^. Women with PCOS have traditionally been viewed as being protected from bone fragility disorders given the positive effects of their higher androgens, insulin and mechanical loading provided by increased body weight on bone. The tendency to increased androgen levels and body weight also leads to greater muscle mass in women with PCOS, which further positively influences bone density and strength^31, 32^. However, PCOS is also related to increased levels of inflammatory mediators, which further add to their risk of other health disorders such as cardiovascular disease^12–14^. In large part due to the assumption of bone protection in women with PCOS, the potential contribution of chronic inflammation in compromising optimal bone health has not been previously investigated.

In this first exploratory study to compare comprehensive measures of bone density and bone strength of women with and without PCOS in relation to their inflammatory, oxidative stress and anthropometric characteristics, we observed that the classical positive predictors of increased bone strength and quality, such as body and muscle mass, were seen in controls but were dissociated in women with PCOS. The mechanism underlying this phenomenon appears to be, at least in part, related to their elevated level of subclinical inflammation, which was the predominant factor in this study that negatively predicted the radial bone strength in women with PCOS, but not in control women.

Notably, there was a complete uncoupling between the radial SSI and the hip BMD measures in women with PCOS, which is in stark contrast to women without PCOS for whom the radial SSI was one of the strongest correlates of hip BMD. This implies that factors influencing bone remodeling, architecture and bone quality are normally linked, but this relationship may be disrupted in women with PCOS. This is further demonstrated by the observation that despite women with PCOS having significantly greater forearm muscle mass, estimated by the MCSA, it did not, as expected, relate to increased bone strength. The strongest negative predictor of radial bone SSI in women with PCOS was inflammation, as measured by CRP/albumin. This finding is in line with the association between high systemic CRP levels and reduced BMD, osteopenia and osteoporosis in otherwise healthy premenopausal and menopausal women^8^.

It is noteworthy that the negative association between inflammation and bone parameters observed here was most apparent in the radius, which is a non-weight bearing bone. Given inflammation was shown to increase collinearly with central adiposity and BMI in women with PCOS, it is probable that any potential negative influence of inflammation on bone health would be partly masked by the dominant influence of adiposity and body mass on a weight-bearing bone such as the hip. Thus, it should be noted that the opposing forces of inflammation and abdominal obesity make it challenging to untangle their effects on bone physiology when evaluating a weight-bearing bone.

The lack of association between our proxy measures for oxidative stress, LTL and urinary 8-OHdG, and any of the bone parameters may suggest that these are not sensitive measures of oxidative stress that impacts bone health in women of this age group. Indeed, the association between LTL and bone health measures has been contradictory and may depend on the population studied^33–35^. Part of the problem is that there is currently no consensus on what measures of oxidative stress are best for assessing their influence bone health^36^. The discrepancy in the literature on this relationship is thought to be, at least in part, attributable to differences in laboratory methods, types of biological specimens used, as well as limitations with respect to controlling for the impact of lifestyle factors such as diet and supplementation^36^.

Of clinical concern, our findings suggest that oral contraceptives, which are commonly used in the treatment of women with PCOS, may warrant additional consideration. Along with central adiposity, our findings suggest that current oral contraceptive use may be independently linked to increased systemic inflammation. This is consistent with studies showing that treatment with oral contraceptives leads to increased CRP levels^37^, and their use in PCOS is associated with lower BMD^38^.

Given the current paucity of published studies on the prevalence of bone fractures in premenopausal women with PCOS, it is challenging to fully assess the clinical implications of our findings. A prospective 21-year follow-up study of menopausal women (aged 61 to 78 years), 25 with PCOS and 68 age-matched controls, found that despite maintaining a higher free androgen index into menopause, 56% of women with PCOS had experienced an incident fracture in contrast to 41% of controls^39^. In contrast, a Danish study found that women with PCOS are at reduced risk of fractures particularly when younger than 30 years^7^. Our study specifically included premenopausal women between 35 and 47 years of age as they would have achieved peak bone mass, and would also have experienced an extended period of exposure to inflammation known to be associated with their condition. Our data suggests that chronic inflammation may take its toll on the bones of women with PCOS, and its effect may eventually erode the bone protective influence of increased mechanical load and androgen excess.

This investigation included a relatively small sample of women, and it requires replication in larger, preferably population-based, cohorts. A larger study would also allow for improved matching for characteristics, including body mass indices and age. Additionally, a larger study would permit the inclusion of other potentially important contributing variables, such as insulin resistance and sex steroids^40^, in the context of subclinical inflammation. PCOS-associated hyperandrogenemia has, however, previously been shown not to directly contribute to the chronic low grade inflammation observed in these women^41^. Since this was a volunteer sample, self-selection bias could have led us to recruit more women with PCOS who were concerned about their bone health; however, we expect this influence would equally affect our control sample.

In conclusion, optimal radial bone strength was attenuated in women with PCOS, in association with subclinical inflammation, which, in turn, was positively predicted by an increased waist-to-height ratio and the current use of oral contraceptives. These findings warrant replication in larger prospective studies. Management of the health issues of women with PCOS should take into account the impact of both the condition and its treatment on long-term bone health.
